# Supplementary material for: Nursing Student Satisfaction with the Teaching Methodology Followed during the COVID-19 Pandemic
Source: Healthcare (Basel). 2022 Mar 22;10(4):597. doi: 10.3390/healthcare10040597 (PMC9032741; doi:10.3390/healthcare10040597)
Supplement: Supplementary file 1 [file healthcare-10-00597-s001.zip › Supplementary Material Healthcare.pdf]

SUPPLEMENTARY MATERIAL

Table S1. Nursing students´ satisfaction by dimensions and scale items with online and bimodal learning measured with adapted CUSAUF questionnaire

**Dimensions and scale items**	**Online learning Mean (SD)**	**B-learning Mean (SD)**	**p**
**Subjects structure**	2.61 (0.70)	3.01 (0.58)	0.001
1. The course's structure, contents and training objectives are in accordance with those established in the academic guide adapted to the new situation	2.77 (0.81)	3.10 (0.63)	0.001
2. The activities performed on the virtual platform have contributed to the understanding and application of the acquired knowledge	2.34 (0.90)	2.90 (0.74	0.001
3. The design and material provided for the subjects and the delivery dates of activities were correctly configured.	2.73 (0.89)	3.05 (0.74)	0.003
**Teacher-related aspects (teaching performance)**	2.74 (0.59)	2.94 (0.54)	0.001
4. Teachers/lecturers presented the adapted contents of subjects clearly and in-depth, and explained them whenever necessary	2.38 (0.88)	2.93 (0.80)	0.001
5. Teachers provided clear guidance about the rules to work on the virtual learning platform before and while subjects were underway	2.61 (0.90)	2.69 (0.90)	0.524
6. Online digital educational resources and tools were suitably used by the teachers	2.66 (0.80)	2.59 (0.81)	0.507
7. Teachers mastered the themes worked on in subjects	2.86 (0.78)	3.18 (0.74)	0.002
8. Teachers correctly answered our concerns, doubts and technical difficulties with using the virtual platform and teaching tools outside class	2.73 (0.88)	2.73 (0.79)	0.990
9. The activities delivered by students were properly marked and obtained suitable feedback.	2.99 (0.82)	3.01 (0.78)	0.871
10. Evaluating or marking course activities was objective and based on the criteria previously set by teachers	2.84 (0.87)	3.17 (0.70)	0.002
11. Teachers' feedback or remarks about students' performed activities were respectful, coherent and based on the previously set evaluation criteria.	2.95 (0.85)	3.17 (0.70)	0.046
12. Teachers encouraged students' participation and their collaborative work during the course by different means (forums, tutoring, etc.)	2.75 (0.85)	2.96 (0.76)	0.053
**ASPECTS RELATED TO THE CONTENTS OF THE DIFFERENT SUBJECTS ON THE WHOLE**	2.60 (0.66)	2.92 (0.54)	0.001
**13.** The contents taught and the employed educational resources (documents, videos, illustrations) were up to date	2.72 (0.89)	3.05 (0.77)	0.003

14. The employed educational resources (documents, videos, illustrations, etc.) were sufficient and suitable for profoundly dealing with the themes proposed during the course.	2.51 (0.89)	3.08 (0.67)	0.001
15. The presented/taught contents were easy to understand.	2.46 (0.79)	2.89 (0.65)	0.001
16. I think that the originality of the offered contents was suitable.	2.38 (0.88)	2.60 (0.75)	0.036
17. I think that the interest of contents from a practical point of view was suitable	2.56 (0.97)	2.89 (0.69)	0.02
18. I think that contents were appropriate interesting.	2.78 (0.89)	3.04 (0.68)	0.012
19. The relation between the objectives and the offered contents was suitable.	2.72 (0.90)	3.05 (0.67)	0.001
20. The relation between the schedule and the offered contents was suitable	2.52 (0.86)	2.82 (0.84)	0.008
21. The evaluation of contents (exams and tests) offered over the platform/s was objective and rigorous	2.73 (0.93)	2.85 (0.87)	0.306
22. I think that the scientific and didactic/educational quality of the offered contents was suitable	2.66 (0.76)	3.00 (0.78)	0.001
**COMMUNICATION-RELATED ASPECTS**	2.91 (0.85)	3.05 (0.63)	0.147
23. I found that communicating with the teacher was easy by means of the available communication tools: email, forum, chat, video conferences, online classes, etc.	2.80 (0.93)	3.00 (0.78)	0.078
24 I found that communicating online with my other classmates in the environment was simple	3.01 (0.94)	3.11 (0.75)	0.400
**ASPECTS RELATED TO THE VIRTUAL LEARNING ENVIRONMENT**	3.05 (0.64)	3.04 (0.56)	0.971
25. The technical functioning in the environment was easy to understand	3.04 (0.79)	2.93 (0.80)	0.305
26. I liked the available platforms/means because they were easy to browse	3.15 (0.81)	3.21 (0.75)	0.523
27. I think that the esthetic quality of the environment (size and type of fonts, colors, etc.) was suitable	3.31 (0.76)	3.21 (0.64)	0.266
28. The suitability of the different esthetic elements on platforms was appropriate (texts, images, graphs)	3.23 (0.76)	3.14 (0.67)	0.329
29. Platforms' response times (time to access a link, to gain access to different tools, etc.) were suitable	2.73 (0.91)	3.01 (0.76)	0.010
30. The environment's technical functioning (connection and work on platforms) was generally suitable	2.80 (0.92)	2.73 (0.83)	0.531
TOTAL SCORE	2.75 (0.56)	2.94 (0.49	0.004
Values are expressed as the mean with standard deviation in brackets and 95% confidence interval.			

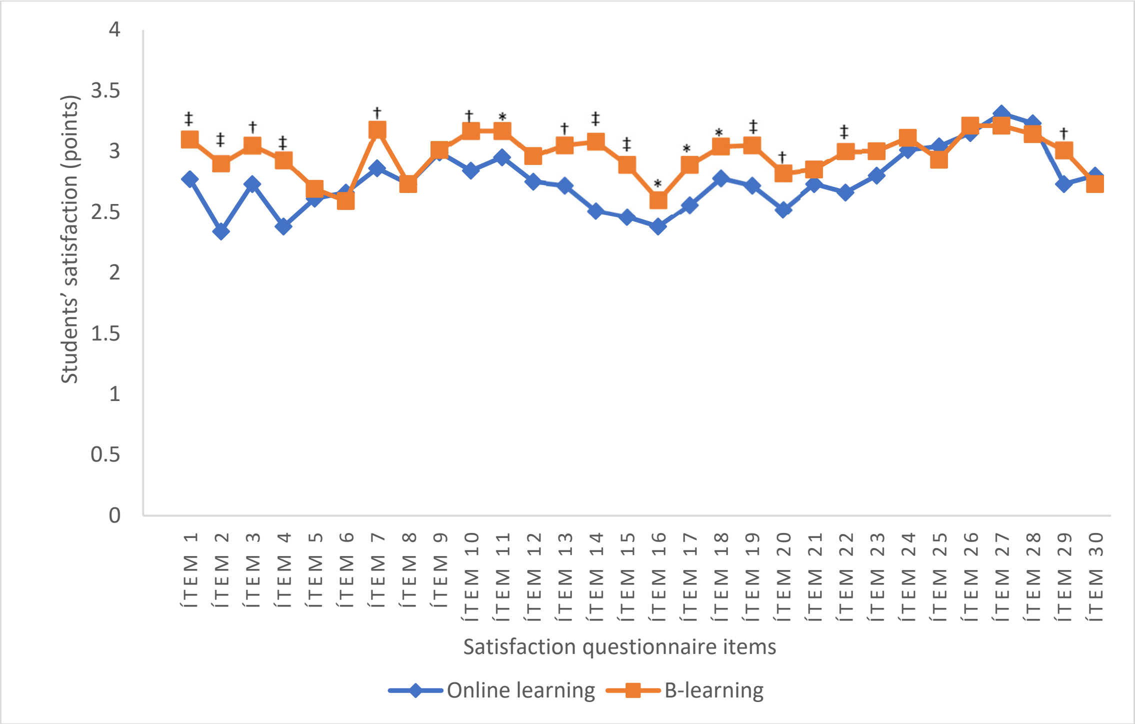

Figure S1. Students' satisfaction according to the survey item and learning methodology. * p<0,05; †p<0,01; ‡ p <0,001
